# Neuroprotective Effects of a Composite Based on Irradiated Gold Nanoparticles and Lipid Vesicles in a Zebrafish Model

**DOI:** 10.3390/pharmaceutics18050585

**Published:** 2026-05-09

**Authors:** Silvia Garofalide, Daniela Angelica Pricop, Eliza Olteanu, Sebastian Emmanuel Pricop, Ion Brinza, Razvan Stefan Boiangiu, Alexandru Cocean, Georgiana Bulai, Elena Laura Ursu, Iuliana Motrescu, Iuliana Cocean, Lucian Hritcu, Silviu Gurlui

**Affiliations:** 1 Laboratory of Applied Meteorology and Climatology, RECENT AIR, Research Center with Integrated Techniques for Atmospheric Aerosol Investigation in Romania, Institute of Interdisciplinary Research, Alexandru Ioan Cuza University of Iasi, a Building, Physics, 11 Carol I, 700506 Iasi, Romania; silvia.garofalide90@gmail.com (S.G.); alexcocean@yahoo.com (A.C.); iuliacocean@gmail.com (I.C.); 2 Atmosphere Optics, Spectroscopy and Laser Laboratory (LOASL), Faculty of Physics, Alexandru Ioan Cuza University of Iasi, 11 Carol I Blvd., 700506 Iasi, Romania; daniela.a.pricop@gmail.com (D.A.P.); sebastianpricop11@yahoo.com (S.E.P.); 3 Laboratory of Astronomy and Astrophysics, Astronomical Observatory, RECENT AIR, Research Center with Integrated Techniques for Atmospheric Aerosol Investigation in Romania, Institute of Interdisciplinary Research, Alexandru Ioan Cuza University of Iasi, Astronomical Observatory, 11 Carol I Blvd., 700506 Iasi, Romania; 4 Physics Department, Physics Faculty, Alexandru Ioan Cuza University, 700506 Iasi, Romania; eliza.olteanu14@yahoo.com; 5 Department of Environmental Sciences, “Lucian Blaga” University of Sibiu, 5–7 Dr. Ion Ratiu Str., 550012 Sibiu, Romania; ion.brinza@ulbsibiu.ro; 6 Department of Biology, Faculty of Biology, Alexandru Ioan Cuza University of Iasi, 700506 Iasi, Romania; razvan.boiangiu@uaic.ro; 7 Integrated Center of Environmental Science Studies in the North Eastern Region (CERNESIM), Department of Exact and Natural Sciences, Institute of Interdisciplinary Research, Alexandru Ioan Cuza University of Iasi, 11 Carol I Blvd., 700506 Iasi, Romania; georgiana.bulai@uaic.ro; 8 Advanced Research Center for Bionanoconjugates and Biopolymers, P. Poni Institute of Macromolecular Chemistry, 700487 Iasi, Romania; ursu.laura@icmpp.ro; 9 Sciences Department, Horticulture Faculty, Ion Ionescu de la Brad Iasi University of Life Sciences, 700490 Iasi, Romania

**Keywords:** Alzheimer’s disease (AD), *Danio rerio*, gold nanoparticles irradiated and modify with lipid, neuroprotection, oxidative stress, scopolamine, memory enhancement, cholinergic system, drug delivery, nanomedicine

## Abstract

**Background**: In this work, gold nanoparticles synthesized by the chemical method were exposed to natural green light, then associated with a lipid layer of phosphatidylcholine by physical adsorption, without excluding their partial encapsulation. **Methods**: A suspension of lipid vesicles grafted with irradiated nanoparticles (Au(ir)L) was obtained that showed improved colloidal stability, evidenced by a higher negative ζ potential (−23.8 mV compared to −17.08 mV for AuL), an increased hydrodynamic size, and a higher lipid coverage, suggesting improved nanoparticle–membrane electrostatic interactions. The biological effects of these vesicles were evaluated in a zebrafish model of scopolamine-induced cognitive impairment. Behavioral and biochemical analyses were conducted to assess their impact on anxiety-like behavior, memory, and oxidative stress, using galantamine as a reference compound. **Results**: Under non-induced conditions, no significant behavioral differences were observed between the control and nanoparticle-treated groups, supporting the biocompatibility of the formulations. In scopolamine-treated zebrafish, both AuL and Au(ir)L showed partial improvements in behavioral parameters; however, these effects were not consistently statistically significant across all endpoints. Notably, more consistent effects were observed at the biochemical level, where both formulations, particularly Au(ir)L, significantly modulated acetylcholinesterase activity and reduced markers of oxidative stress, including lipid peroxidation. **Conclusions**: Overall, these findings suggest that lipid-grafted gold nanoparticles, especially in their irradiated form, exhibit moderate neuroprotective potential, primarily supported by biochemical outcomes and accompanied by partial behavioral improvements.

## 1. Introduction

Even though gold nanoparticles (AuNPs) are generally considered non-toxic and biologically inert, they can induce toxic responses depending on their size, shape, surface charge, and the nature of the coating material [1]. The cellular internalization of AuNPs depends strongly on cell type and nanoparticle properties. In the absence of active targeting, gold nanoparticles predominantly interact with cell membranes through adsorption or passive uptake mechanisms [2]. Several studies indicate that nanoparticle–membrane interactions are governed by surface charge, size, and coating, with positively charged nanoparticles generally exhibiting stronger interactions with lipid bilayers. However, direct membrane penetration is not universal and depends on physicochemical context, with adsorption and membrane association often being the dominant processes. A growing strategy to improve nanoparticle biocompatibility involves their association with lipid bilayers, which can occur through surface adsorption, partial insertion, or encapsulation. These interactions can reduce opsonization, enhance colloidal stability, and improve biological compatibility. Because it mimics human cell membranes, a lipid coating improves NP stability because it increases electrostatic repulsion, reducing the tendency for aggregation and thus reducing cytotoxic effects [3]. Previous studies have shown that the size of nanoparticles is crucial in the interaction with the lipid bilayer. The nanoparticles with sizes ranging from (25–35 nm) were adsorbed on the outer surface of the membrane, while larger Au nanoparticles (50–60 nm) were poorly adsorbed. Vesicular internalization was achieved for Au nanoparticles with sizes ranging from (5–10 nm) because they exhibit a higher curvature, requiring lower penetration energy [4]. When interacting with lipid vesicles, nanoparticles may associate with the membrane surface via electrostatic and van der Waals interactions, but may also partially insert into or become entrapped within the lipid bilayer depending on their surface chemistry and preparation conditions. Another advantage of coating AuNPs with lipids is the prevention of the formation of hot spots through electrostatic repulsion, thus ensuring their good dispersion [5]. However, there are limitations because it is known that the citrate layer on the surface of nanoparticles exerts a strong resistance to ligand exchange [6]. Solutions are needed that produce oxidations in the citrate layer and facilitate the interaction of nanoparticles with the lipid membrane. A solution proposed in this work is to modify the level of interaction of AuNPs with the lipid bilayer by irradiating the surface of the nanoparticles with visible light. It is known that the optical properties of AuNPs are controlled by the collective oscillation of conduction electrons, which results from the interaction with the electric field of the incident light [7]. The electric field of the incident radiation creates a strong dipole electric field inside the metallic nanostructures, which creates a force that tries to compensate for the difference between the two fields, resulting in a unique resonant wavelength [7]. When electrons in the conduction band resonate with the frequency of the incident visible light, the nanoparticles absorb the light and enter into coherent oscillations. A heated electron gas will form, which will give off heat to the nanoparticle network. In turn, the metallic structure will give up energy to neighboring molecules through a photoexcitation process, causing various physical or chemical changes [6].

Alzheimer’s disease (AD) is a progressive neurodegenerative disorder characterized by cognitive decline, cholinergic dysfunction, and increased oxidative stress. Despite extensive research, current therapeutic strategies provide only symptomatic relief, highlighting the need for novel approaches targeting multiple pathological mechanisms. Nanoparticle-based systems have emerged as promising tools in this context, particularly due to their ability to modulate oxidative stress, improve drug delivery, and interact with neuronal membranes. In this regard, lipid vesicles grafted with gold nanoparticles represent a potential multifunctional platform, combining the physicochemical stability of metallic nanoparticles with the biocompatibility of lipid systems, which may enhance neuroprotective effects [8]. It has been reported that (AuNPs) can reduce amyloid aggregation in neurodegenerative diseases. They act by binding to amyloid-beta (Aβ) peptides, inhibiting their conversion from soluble monomers to toxic β-sheet-rich fibrils, leading to neuroprotection [9]. They can also act as stable antioxidants by scavenging free radicals and inhibiting the formation of (ROS) through surface catalytic activity [10]. In addition, visible light irradiation of citrate-stabilized Au nanoparticles has previously been proposed as a mean of partially oxidizing the citrate coating, thus modulating the surface reactivity of the nanoparticles and improving the interaction with lipid membranes. Structural characterization highlighted several differences between irradiated and non-irradiated AuNP conjugates, which could explain both stability and antitumor activity. As drug carriers, they have the potential to avoid systemic toxicity in healthy tissues [11]. The pathogenesis of the disease is multifaceted, involving elements such as the aggregation of beta-amyloid (Aβ) and tau proteins [12], acetylcholine (ACh) deficits, neuroinflammatory processes, oxidative stress, disruptions in metal homeostasis, insulin resistance, as well as dysfunctions in mitochondrial and autophagic mechanisms [13]. These elements play a critical role in diagnosing, monitoring disease progression, and formulating targeted therapeutic interventions. Despite the utility of biomarkers in early detection and treatment efficacy assessment, the development of effective therapeutic strategies for AD continues to present significant challenges.

To evaluate the neuroprotective potential of these lipid vesicles grafted with gold nanoparticles, an appropriate in vivo model is required. The zebrafish (*Danio rerio*) has emerged to be a robust and translational model in neuroscience, widely used in the study of the pathological and pharmacological aspects of AD due to its numerous advantages. Importantly, this model enables the simultaneous assessment of behavioral, biochemical, and oxidative stress parameters, making it particularly suitable for evaluating multifunctional nanomaterials such as lipid–gold nanoparticle systems in the context of neurodegenerative disorders. It exhibits significant genetic similarity to humans, with approximately 70% of its genes being shared, and 84% of the genes involved in human diseases are present in its genome [8,12,13]. These aspects allow for precise genetic manipulation and the development of transgenic models that replicate pathological features of AD, such as amyloid plaques and neurofibrillary degeneration [14]. Furthermore, the neuroanatomical structures and neurochemical pathways of the zebrafish are comparable to those of the human brain, making it possible to study the neurological changes associated with AD [15]. At the same time, zebrafish exhibit behaviors similar to those of humans, such as anxiety, learning, and decision-making. Additionally, the regulation of sleep and circadian disorders observed in this model provides valuable insights into the impact of AD on cognitive functions. Recent studies have demonstrated the potential of this model in aging research, providing new insights into cognitive decline and characteristics associated with AD [16]. At the same time, testing chemical compounds on zebrafish is fast and efficient, thanks to the ability to administer drugs directly into the water and monitor the effects in real-time [17]. Thus, the zebrafish has established itself as a versatile and promising experimental model, capable of accelerating the understanding of the molecular mechanisms of AD and facilitating the development of innovative therapies. It represents a valuable resource for future research in the field of neurodegeneration [18].

Therefore, the present study aimed to investigate the effects of lipid vesicles grafted with irradiated and non-irradiated AuNPs, with particular emphasis on their interaction with lipid membranes and their neuroprotective potential. Given that both adsorption onto the vesicle surface and partial incorporation within the lipid bilayer may occur, this work evaluates how these interactions influence nanoparticle stability and biological activity in a zebrafish model of scopolamine-induced cognitive impairment.

## 2. Materials and Methods

The reagents used were purchased from Sigma-Aldrich Chemical Co., Steinheim, Germany: hydrated auric tetrachloride (HAuCl_4_ 3H_2_O), sodium hydroxide (NaOH), and sodium citrate dihydrate (C_6_H_9_Na_3_O_9_). Also, Lα phosphatidylcholine from soybean Type IVS ≥ 30% and chloroform were acquired from Sigma-Aldrich, Chemical Co, Steinheim, Germany. All solutions were prepared with deionized and distilled water (18.2 MΩcm) obtained through a purification and deionization system produced with Barnstead EasyPureII Milli-Q, Thermo Fisher Scientific, Waltham, MA, USA. A Sigma D6191-25EA dialysis tube, Life Science of Merck KGaA, Darmstadt, Germany, with a pore size of 12,000 Da was used for dialysis of the samples.

The green light with which the AuNPs were irradiated was obtained by placing a green photographic filter in front of a beam of incoherent light produced by a halogen lamp.

### 2.1. Vesicle Preparation

The AuNPs were prepared using a modified version of the Turkevich method in which sodium citrate plays the role of both the reactant and stabilizer of the nanoparticles. Several work steps were required to obtain lipid vesicles grafted with gold nanoparticles.

Stage 1. In a glass flask, 5 mL of chloroauric acid dissolved in water with a concentration of 25 mM was mixed with 16.5 mL of NaOH (20 mM). The volume was then brought to 47 mL by adding water. The flask was placed in a water bath at a temperature of 100 °C under magnetic stirring conditions of 800 rot/min for 30 min until thermodynamic equilibrium was established. Then, 1.5 mL of sodium citrate (50 mL/mg) was added, and the magnetic stirring process continued for another 15 min at a temperature of 85 °C. The suspension changed its color to purple-red. The flask was stored cold at 4 °C to stop the further nucleation process. At this stage, the reduction of auric chloride occurred with the formation of gold nanoparticles coordinated with citrate groups [19].

Stage 2. A volume of 10 mL of AuNP suspension was placed in a Petri dish and exposed to a 50 W halogen lamp positioned at a height of 24.5 cm above the sample surface. The sample (Au(ir)L) was irradiated using a 520 nm green photographic filter. The optical energy and the intensity of the light absorbed by the irradiated sample were monitored using a Solar Light PMA 2100 radiometer, Solar Light Company Inc, Glenside, PA, USA, equipped with a PMA 2130 sensor. The source emission energy was 20.6 W/m^2^, and the absorbed energy was 3.22 kJ/m^2^. The sample was irradiated for 80 min in a series of 10 min of light exposure and 5 min of rest, in order to maintain the suspension temperature constant at 23 °C during the 10 min of irradiation. The method is according to Lupusoru et al. (2020) [19].

The following samples were prepared:

AuL: A quantity of 0.011 g lipid was dissolved in 1 mL chloroform in a vessel, which was placed in a high vacuum oven for 2 h to remove the chloroform by evaporation.

The dried lipid film was then hydrated with 16 mL of AuNP suspension (0.25 mM) and then subjected to a probe-type sonicator (Bandelin electronic D-12207, Berlin, Germany) for 20 min at 25%.

Au(ir)L: A quantity of 0.011 g lipid was dissolved in 1 mL chloroform in a vessel, which was placed in a high vacuum oven for 2 h to remove chloroform by evaporation. The dried lipid film was then hydrated with 16 mL of Au(ir)NP suspension (0.25 mM) and then sonicated for 20 min at 25%.

All samples were dialyzed using a 12,000 Da MWCO membrane in distilled water for 2 h to remove reactants, free polymer from suspensions (unreacted sodium citrate, NaOH, and residual HAuCl_4_), and to adjust the pH to 6. The pH of 6 was confirmed by pH meter measurement after dialysis. All synthesis steps, irradiation, and composite preparation were performed in three independent batches to confirm reproducibility.

#### Characterization of Vesicles

Spectrophotometric measurements were performed using a Shimadzu UV-1800 spectrophotometer (Kyoto, Japan) in the wavelength range of 200–700 nm, with a scan rate of [800 nm/min], a slit width of [1 nm], and quartz cuvettes with a path length of 1 cm. All spectra were recorded at room temperature (23 °C). Hydrodynamic size distribution measurements were performed by dynamic light scattering (DLS) using the Zetasizer Nano ZSP instrument, Malvern Instruments, Tokyo, Japan, and the average values were obtained for 3 consecutive measurements. Micrographic analyses were performed by transmission electron microscopy (TEM) using the Philips CM100 Device, Philips Electron Optics, Eindhovento, Netherlands, to obtain information about the morphology, size, and dimensional distribution of the gold nanoparticles. Images were taken by dark field (DF) optical microscopy with a 40× oil immersion objective OPTIKAB-383DK, Bergamo, Italy. The images were processed using ImageJ software version 1.54s to obtain the distribution of AuNPs and AuNP-grafted vesicles. Surface chemical analysis of the samples was performed using a Bomem MB154S FTIR spectrometer at an instrumental resolution of 4 cm^−1^ (Bomem, ABB group, Québec, QC, Canada). Surface analysis of the samples was performed by scanning electron microscopy (SEM). This was performed using a Quanta 450 from FEI ThermoScientific, Brno, Czech Replublic, on samples pipetted directly onto carbon-coated aluminum stubs. Low vacuum conditions (100 Pa) in a water vapor atmosphere with an electron beam accelerated at 10 kV were used.

### 2.2. In Vivo Test

A total of 48 adult zebrafish (*Danio rerio*), aged 5 months and evenly distributed by sex (50% males, 50% females), were obtained from the European Zebrafish Resource Center (Institute of Toxicology and Genetics, Karlsruhe, Germany). The fish were acclimated for two weeks in a 70 L tank under standard laboratory conditions, including continuous aeration and a 14:10 h light–dark cycle starting at 08:00. Environmental parameters were maintained as follows: temperature 28 ± 0.5 °C, pH 7.0 ± 0.15, dissolved oxygen 6.0 ± 0.1 mg/L, ammonia < 0.01 mg/L, total hardness 6 mg/L, and alkalinity 22 mg/L CaCO_3_. Dechlorinated tap water, treated with Tetra AquaSafe (Tetra GmbH, Melle, Germany) to neutralize chlorine, chloramine, and heavy metals, was used to ensure optimal water quality, which remained stable throughout the study.

Fish were fed twice daily (09:00 and 15:00) with commercial NovoMalawi flakes (JBL, Neuhofen, Germany). Feeding was calibrated so that food was consumed within 10 min, during which aeration was temporarily suspended. All procedures were conducted following the ARRIVE guidelines and complied with the European Directive 2010/63/EU for the protection of animals used for scientific purposes and were approved by the Animal Ethics Committee of the Faculty of Biology, Alexandru Ioan Cuza University of Iasi, Romania (approval no. 1714, date of approval 6 July 2023). Efforts were made to minimize both the number of animals used and any potential suffering.

#### 2.2.1. Zebrafish Treatment

Stock solutions of 1 mg/mL of GAL (Sigma Aldrich, Darmstadt, Germany) were prepared in distilled water. SCOP solution (100 μM, Sigma Aldrich, Darmstadt, Germany) was prepared in 2 L of distilled water, and to mitigate variables that could affect behavior, this solution was prepared before application and replenished after each exposure session. Following SCOP exposure, fish were transferred to fresh treatment solutions, thereby minimizing the possibility of direct physicochemical interaction between SCOP and the nanoparticle formulations. Subjects were kept in a 10-L tank and segregated with 6 individuals per group into 4 distinct experimental cohorts, where two cohorts were administered SCOP, while the other two cohorts did not receive SCOP, adhering to subsequent classification: Cohort 1 comprised 4 experimental groups containing non-SCOP nanoparticles (1. CTR;2. GAL;3. AuL;4. Au(ir)L); Cohort 2 consisted of 4 experimental groups comprising nanoparticles that were initially subjected to SCOP pretreatment (1. CTR; 2. GAL; 3. AuL; 4. Au(ir)L). Animals were randomly allocated to experimental groups using a computer-generated randomization sequence to minimize selection bias. Sample size determination was conducted using InVivoStat 4.7, an R-based statistical software package. Based on a significance level (*p*) of 0.05, a sample size of *n* = 10 zebrafish per group provided a statistical power of 98% to detect a biologically relevant effect size of 20%.

**Figure 1:**
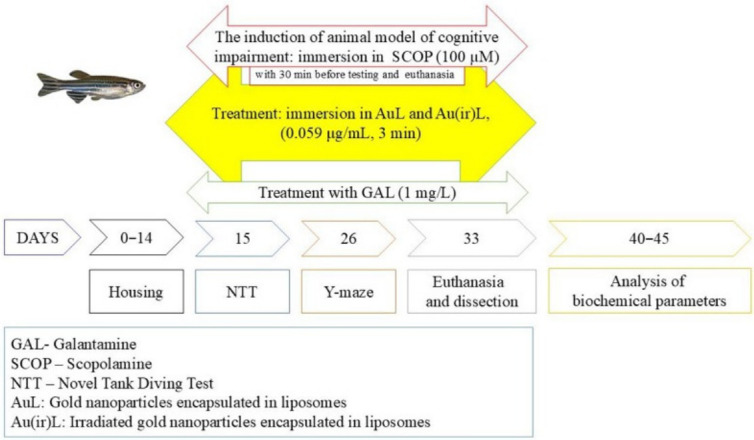
Schematic illustration outlining the methodological framework of the study.

Before performing the behavioral tasks, all groups assigned to scopolamine treatment, including the SCOP control group (CTR + SCOP), were subjected to individual immersion in the SCOP solution (100 μM) for 30 min. The untreated control group (CTR) was not exposed to SCOP [20]. Subsequently, each specimen was treated individually for 3 min in 0.5 L glass containers containing 0.059 μg/mL of (AuL, Au(ir)L). Before being placed into the testing tank, the fish were given a 5-min rest period in their respective baseline tanks. GAL was acutely administered at a concentration of 1 mg/L for 3 min prior to the initiation of the experimental protocol (Figure 1).

#### 2.2.2. Behavioral Activity Assessment

Zebrafish behavior was recorded using a Logitech HD Webcam C922 Pro Stream (Logitech, Lausanne, Switzerland), which provided high-resolution video suitable for detailed behavioral analysis. The recorded videos were subsequently analyzed using ANY-maze^®^ software (version 7.48; Stoelting Co., Wood Dale, IL, USA), a validated platform for automated tracking and behavioral assessment in animal studies. Behavioral assessments were performed by investigators blinded to the treatment groups to reduce detection bias.

#### 2.2.3. Novel Tank Diving Test (NTT)

In the NTT, zebrafish display distinct behavioral responses to the anxiety elicited by exposure to a new environment. The protocol employed in this study was adapted from Cachat et al. [21]. The testing apparatus consisted of a trapezoidal glass tank filled with 1.5 L of water sourced from the fish’s home tank to minimize additional stress. The tank dimensions were as follows: bottom length 23.9 cm, top length 28.9 cm, height 15.1 cm, width at the top 7.4 cm, and width at the bottom 6.1 cm. Each fish was tested individually, and its behavior was recorded for 6 min using a webcam positioned 40 cm in front of the tank. The tank was virtually divided into two equal horizontal zones (top and bottom), each representing 50% of the total water column height. The top zone corresponded to the top half of the tank, while the lower zone represented the bottom half. This delimitation was implemented within the ANY-maze^®^ tracking system to ensure consistent and automated behavioral quantification across all experimental groups. Anxiety-like behavior and locomotor activity were evaluated using established behavioral parameters previously described in the literature [22] including latency to enter the top zone (s), time spent in the bottom zone (s) (an indicator of anxiety), distance traveled in the top (m), total distance traveled (m), freezing duration (s), and average swimming velocity (m/s).

#### 2.2.4. Y-Maze

Zebrafish memory performance and response to novelty were evaluated using the Y-maze task, following the protocol described by Cognato et al. [23]. The test was conducted in a Y-shaped tank with three arms (25 × 8 × 15 cm), filled with 3 L of water from the animals’ home tank. Visual cues in the form of distinct geometric shapes (e.g., triangles, circles, and squares) were affixed to the outer walls of each arm to facilitate spatial orientation. The arms of the maze were defined as follows: start arm (A), where the fish began exploration; other arm (B), accessible throughout the experiment; and novel arm (C), which was blocked during the training phase by a transparent glass folding door and opened only during the testing phase. The central zone of the maze was excluded from analysis to avoid ambiguity in arm entries and improve data reliability. The test consisted of two phases separated by a 1-h interval: (1) Training session—the fish was allowed to explore arms A and B for 5 min, while arm C remained closed; (2) Test session—one hour later, the fish was reintroduced into arm A and allowed to freely explore all three arms for 5 min. Four behavioral variables were recorded to assess recognition memory, locomotor activity, and exploration strategy, as outlined by Cognato et al. [22]. Time spent in the novel arm (% of total time) reflects recognition memory and exploratory response to novelty.

1. Total distance traveled (m): serves as an indicator of general locomotor activity and possible anxiety-related changes in behavior.
2. Turn angle (°): measures the angular changes in swimming trajectory, providing insight into spatial exploration patterns.
3. Spontaneous alternation (%): represents the percentage of consecutive entries into three different arms without repetitions, reflecting working memory and navigational strategy.

#### 2.2.5. Biochemical Parameters Analysis

Following completion of the behavioral assessments, zebrafish were anesthetized via immersion in ice-cold water (2–4 °C) for 10 min, followed by rapid euthanasia, as previously described by Brînza et al. [20]. Brains were carefully dissected, weighed, and immediately stored at −20 °C for subsequent biochemical analysis. On the following day, brain tissues were homogenized in ice-cold 0.1 M potassium phosphate buffer (pH 7.4) containing 1.15% KCl at a 1:10 (*w*/*v*) ratio using a bead mill homogenizer. The homogenates were centrifuged at 14,000 rpm for 15 min at 4 °C, and the resulting supernatants were collected for biochemical assays. Biochemical analyses were performed by investigators blinded to the treatment groups to reduce detection bias.

#### 2.2.6. Determination of AChE Activity

The Ellman method [23] was used to measure acetylcholinesterase (AChE) activity in the brain samples. A reaction mixture of 600 µL was prepared with sodium phosphate buffer, 5,5′-dithiobis-2-nitrobenzoic acid (DTNB), enzymatic extract, and acetylthiocholine chloride (ATCh), as previously described. After 10 min, the reaction was stopped with acetone, and the 2-nitro-5-thiobenzoate anion formed was monitored at 412 nm. AChE activity was expressed in nmol of ATCh hydrolyzed per min per mg of protein, with protein concentration determined using the Bradford method [24].

#### 2.2.7. Determination of SOD Activity

In this study, superoxide dismutase (SOD) activity was assessed using a method established by Winterbourn et al. [25], which measures the enzyme’s ability to inhibit the reduction of nitroblue tetrazolium (NBT) by superoxide radicals. The reaction mixture included 0.067 M potassium phosphate buffer, enzyme extract, 0.1 M EDTA solution, 0.12 mM riboflavin solution, and 1.5 mM NBT solution. Absorbance was measured at 560 nm, and SOD activity was expressed in enzyme units per mg of protein, with protein concentration determined using the Bradford method [26].

#### 2.2.8. Determination of CAT Activity

Catalase (CAT) activity was quantified using the colorimetric method of Sinha [26]. In summary, 125 μL of the enzyme extract underwent a reaction with an equivalent volume of 0.16 M hydrogen peroxide (H_2_O_2_) for 3 min at 37 °C. The enzymatic reaction was then terminated by adding 500 μL of potassium dichromate and acetic acid reagent, followed by incubation of the resulting mixture at 95 °C for 15 min. The green chromaticity, indicative of chromic acetate, was measured at a wavelength of 570 nm. One unit of CAT activity was defined as the consumption of 1 µmol of H_2_O_2_ within the 3-min period. Enzyme activity was expressed in terms of units of catalase per mg of protein, with protein concentration determined using the Bradford method [26].

#### 2.2.9. Determination of GPX Activity

The methodology outlined by Fukuzawa and Tokumura [27] was used to evaluate glutathione peroxidase (GPX) activity in the present investigation. This protocol is based on the enzymatic catalysis of hydrogen peroxide (H_2_O_2_) breakdown by GPX, using glutathione (GSH) as a reducing agent, which facilitates the generation of oxidized glutathione (GSSG) and water. In 1.5 mL tubes, 78 μL of enzyme extract, 475 μL of 0.25 M sodium phosphate buffer (pH 7.4), 36 μL of 25 mM EDTA solution, and 36 μL of 0.4 M sodium azide (NaN_3_) solution were carefully combined. The samples were incubated for 10 min at 37 °C, after which 50 μL of 50 mM GSH solution and 36 μL of 50 mM H_2_O_2_ solution were added and left to react for another 10 min at 37 °C. The enzymatic reaction was terminated by adding 730 μL of 7% metaphosphoric acid solution, followed by centrifugation of the tubes for 10 min at 14,000 rpm. After centrifugation, 100 μL of the supernatant was transferred to new tubes, to which 1270 μL of 0.3 M disodium phosphate solution and 136 μL of 0.04% 5,5′-dithiobis(2-nitrobenzoic acid) (DTNB) solution were added. Ten minutes after pipetting, absorbance was measured at 412 nm, using a control mixture composed of 100 µL of distilled water, 1270 µL of 3 M disodium hydrogen phosphate (Na_2_HPO_4_) solution, and 136 µL of 0.04% DTNB solution. The specific activity of GPX was calculated by expressing enzyme units (EU) relative to the protein concentration, which was quantified using the Bradford assay [24].

#### 2.2.10. Determination of GSH Content

To determine the total glutathione (GSH) content in brain samples, we used the refined protocol by Salbitani et al. [28]. A volume of 200 µL of supernatant was combined with 1100 µL of 0.3 M disodium phosphate and 130 µL of 0.04% DTNB. The resulting mixture was incubated for 2 min at room temperature, and the yellow chromophore produced was monitored at a wavelength of 412 nm. The quantified amount was expressed as picograms of GSH per milligram of protein, which was quantified using the Bradford assay [24].

#### 2.2.11. Determination of Carbonylated Protein Levels

To assess the concentration of carbonylated proteins, the methodology of Oliver [29], as refined by Ohkawa et al. [26], was used. In the experimental protocol, 1 mg of protein was precipitated using 20% trichloroacetic acid (TCA) and then centrifuged at 14,000 rpm for 5 min. After removing the supernatant, the resulting pellet was reconstituted in 0.2% DNPH (dissolved in 2N HCl) and then precipitated again with 20% TCA. The tubes underwent further centrifugation at 14,000 rpm for 5 min, after which the pellet was washed three times with a solvent mixture of ethanol and ethyl acetate (1:1). The samples were allowed to air dry at room temperature for 10 min, and the pellet was then solubilized in 6 M guanidine hydrochloride (prepared in 20 mM monopotassium phosphate). Absorbance was measured at 370 nm, and the carbonylated protein content was quantified as nmol of DNPH per mg of protein, with protein concentration determined using the Bradford assay [23].

#### 2.2.12. Determination of MDA Levels

The evaluation of malondialdehyde (MDA, lipid peroxide) concentrations in the zebrafish brain was performed following the methodology outlined by Ohkawa et al. [30]. This approach relies on the reactivity of lipid peroxides present in animal tissues with thiobarbituric acid, resulting in the formation of a pink chromophore, which is quantitatively analyzed at a wavelength of 532 nm.

#### 2.2.13. Statistical Analysis

All data are expressed as the mean ± standard error of the mean (SEM). Statistical analyses were performed using GraphPad Prism software (version 9.0, GraphPad Software, San Diego, CA, USA). Before analysis, data distribution was assessed for normality using the Shapiro–Wilk test, and homogeneity of variances was evaluated using Levene’s test. Differences among multiple independent groups were analyzed using one-way analysis of variance (ANOVA), as the experimental design involved a single independent variable (treatment condition) with multiple levels (CTR, GAL, AuL, Au(ir)L, with and without SCOP). When a significant main effect was detected, Tukey’s post hoc test was applied for pairwise comparisons while controlling for multiple testing. A *p*-value of less than 0.05 was considered statistically significant. The sample size for each group is indicated in the corresponding figure legends.

## 3. Results

### 3.1. Characterization of Vesicular Systems

#### 3.1.1. Spectrophotometric Analysis

The samples were subjected to spectral analysis in the range of 200–700 nm under the same physical conditions and at the same concentrations for both types of vesicles.

The values in Table 1 represent three consecutive technical measurements of the same sample.

**Figure 2:**
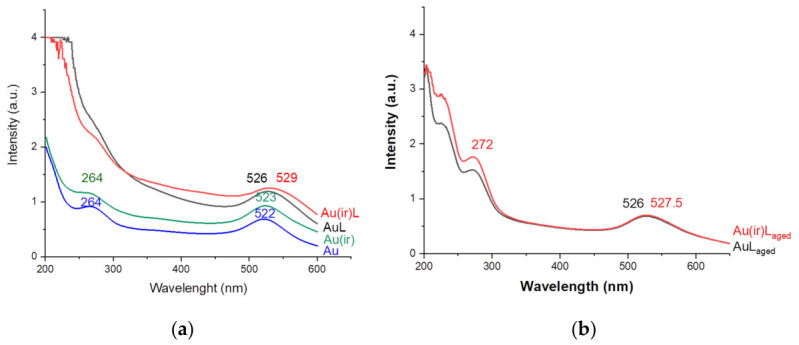
Spectrophotometric analysis of the (**a**) Au, Au(ir), AuL and Au(ir)L, and (**b**) of Au(ir)L _aged_ and AuL _aged_ samples.

According to the spectral analysis, differences were recorded between the gold nanoparticles irradiated with green light and the non-irradiated ones. The Au(ir) sample showed an LSPR band with a higher intensity compared to the Au sample, as shown in Figure 2. This increase in the band intensity is due to the fact that the dipolar oscillation of the electrons has entered into resonance with the incident green light, the specific frequency of which corresponds to the size and shape of the particles. In addition, a slight red shift of the band was also observed due to the rearrangement of the photooxidized citrate layer on the surface of the irradiated nanoparticles [31]. Specifically, the irradiated Au(ir) sample showed a maximum at 523 nm, while the non-irradiated Au sample showed a maximum at 522 nm. As expected, the interaction of the nanoparticles with the lipid layer led to a shift in the LSPR peak to longer wavelengths compared to the individual nanoparticles. The maximum of the AuL sample shifted to 526 nm, while the maximum of the Au(ir)L sample shifted to 529 nm. A sharp increase in the intensity of the absorption peak was observed in both the AuL and Au(ir)L samples compared to the no vesicles samples. According to other studies, the increase in the LSPR signal can be attributed to the formation of a water layer trapped between the nanoparticle surface and the lipid bilayer, whose refractive index is lower than that of the lipid [32]. The concentrations of Au and Au(ir) were calculated according to the Beer–Lambert law for both the Au and Au(ir) samples, as well as for the AuL and Au(ir)L samples:

(1) $$C(AuNP)=\frac{A}{\varepsilon \times l}$$

where ε = 2.01 × 10^8^ M^−1^cm^−1^ L =1 cm.

Both samples showed the characteristic peak of the surface plasmon resonance in the range of 520–525 nm, confirming the formation of spherical AuNPs with an estimated diameter between 13–18 nm. According to the empirical law of Haiss [33], the peak at 522–523 nm corresponds to a size of ~15 nm. The extinction coefficient used (ε = 2.01 × 10^8^ M^−1^·cm^−1^) corresponds to AuNPs of approximately 15 nm diameter, consistent with the peak position at 522–523 nm and the size determined by TEM (~15–16 nm), confirming its appropriateness for concentration calculations as per Haiss et al. (2007) [33].

The concentrations of AuL and Au(ir)L samples were calculated from the absorption spectra obtained after dialysis. Since the dialysis membrane has 12,000 Da, it can completely retain AuNPs (d~15 nm), and the absorbance in graph 2 represents both free nanoparticles and those associated/loaded in vesicles. It can be noted that the apparent concentration increased following dialysis and lipid association, attributed to plasmonic coupling (LSPR coupling) and the change in the refractive index due to the lipid membrane. Consequently, the ratio of AuL or Au(ir)L absorbance to that of bare AuNPs cannot be interpreted as a conventional encapsulation or association efficiency, as both adsorbed and potentially encapsulated AuNPs contribute to the optical signal, and both free and vesicle-associated NPs co-exist in all samples [34]. Absorption spectra were also obtained for the samples aged for 5 months according to Figure 2b. The reduction in the absorbance of the absorption spectra by aging to ~50% compared to fresh samples indicates a partial loss of colloidal stability and a progressive disassembly of the AuNP vesicles over time [34]. A slight blue shift of the aged Au(ir)L absorbance from 529 to 527 nm may indicate a change in the dielectric environment around the particles due to lipid membrane degradation/reorganization. Also, the redefinition of the SPR peak in the aged samples compared to the broader spectrum in the fresh samples may indicate a narrower size distribution of particles that remained in stable suspension, while large particles and aggregates had sedimented compared to the blank Au and Au(ir) samples, whose stability was maintained for more than a year [19]. By comparing the absorbance/concentration values with those of the fresh samples, a relative retention efficiency of AuNPs on vesicles after aging can be estimated.

$$Retention\;\left(\%\right)=\frac{Cage}{Cfresh}\times 100$$

Therefore, we can say that the retention rate of nanoparticles in the Au(ir)L sample is 59.9%, slightly higher than in the AuL sample, where the retention rate is 42.8%. This proves that irradiation moderately improves the long-term stability of the AuNP suspension.

#### 3.1.2. XRD Analysis

**Figure 3:**
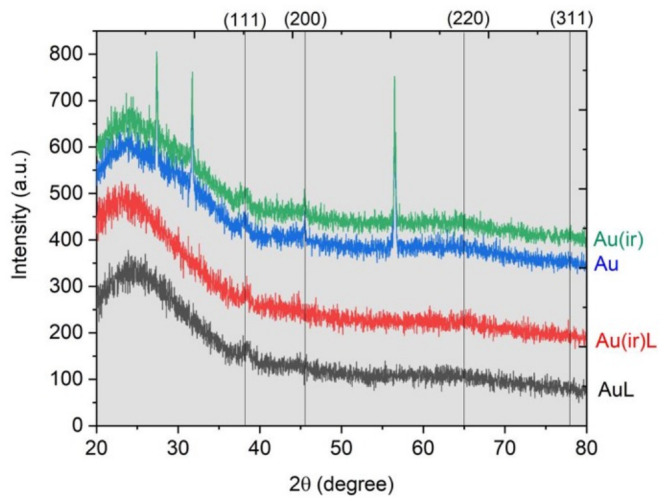
Diffractograms of the Au, Au(ir), AuL, and Au(ir)L samples.

The diffractograms resulting from the XRD analysis (Figure 3) showed nanocrystalline face-centered cubic (fcc) metallic gold in all samples. The sharp peaks around 2θ ≈ 38.26°, 44.48°, 65.18°, and 77.8° corresponded to the (111), (200), (220), and (311) planes of fcc gold, which is considered the typical structure of Au nanoparticles both in unirradiated and irradiated form (according to JCPDS file No. 04-0783). The crystal size calculated using the Scherrer equation for the Au and Au(ir) samples was 15.2 nm for the non-irradiated nanoparticles and 15.4 nm for the irradiated nanoparticles. The shape and the peaks were almost identical for Au, Au(ir), AuL, and Au(ir)L, indicating that irradiation and binding to phosphatidylcholine vesicles do not change the crystalline phase of gold. The peaks assigned to NaCl were present only in undialyzed Au and Au(ir) suspensions and indicate the presence of NaCl formed during the synthesis of the nanoparticles. The suspensions in the AuL and Au(ir)L samples were purified by dialysis, and the NaCl peaks were no longer observed.

In Figure 3, the XRD patterns of the dialyzed AuL and Au(ir)L samples showed that the NaCl peaks were missing compared to the non-dialyzed Au and Au(ir) samples. This analysis confirmed the removal of unreacted sodium citrate, NaOH, and residual HAuCl_4_.

#### 3.1.3. TEM Electron Microscopy Analysis

**Figure 4:**
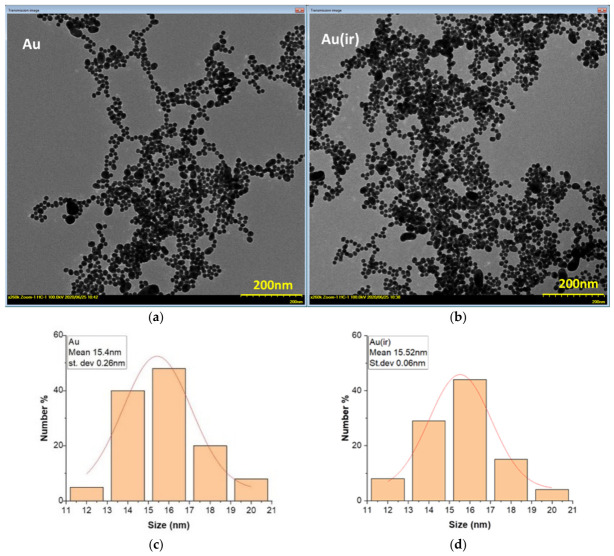
TEM images of the Au sample (**a**) and Au(ir) sample (**b**) and the histograms of the dimensional distributions of the Au sample (**c**) and the Au(ir) sample (**d**). The red line represents the fitting of the distribution graphics.

TEM microscopy and optical microscopy analyses in DF (dark field) were performed to highlight both the average size of the non-irradiated and irradiated nanoparticles and their average degree of lipid coverage (Figure 4).

TEM micrographs showed that there were no significant differences in size between the two samples Au and Au(ir), according to the dimensional histograms made using the ImageJ program. For the Au and Au(ir) samples, average sizes of 16.43 ± 0.02 nm were obtained for 123 measured nanoparticles (Figure 4c), and 15.52 ± 0.06 nm for 100 measured particles (Figure 4d), excluding particles that by overlapping led to dimensional changes. TEM images at higher magnifications could not be obtained for the AuL and Au(ir)L samples because both showed a high level of dispersion.

#### 3.1.4. Darkfield Microscopy Analysis Pdf

In dark field microscopy, the nanoparticles are excited in white light. The dark field condenser emits and focuses a very narrow beam of light onto the sample, with the central part blocked. The objective will collect only the light scattered by the sample, and the image of the bright object on a dark background will be obtained. Nanoparticles appear in a bright color due to the strong scattering of light that creates a bright halo effect called plasmon, which depends on the size and shape of the particles [35]. Through dark-field optical microscopy, the plasmonic formation (bright spot) of the NPs was observed, which was of 2 orders of magnitude larger than the actual size observed in TEM.

**Figure 5:**
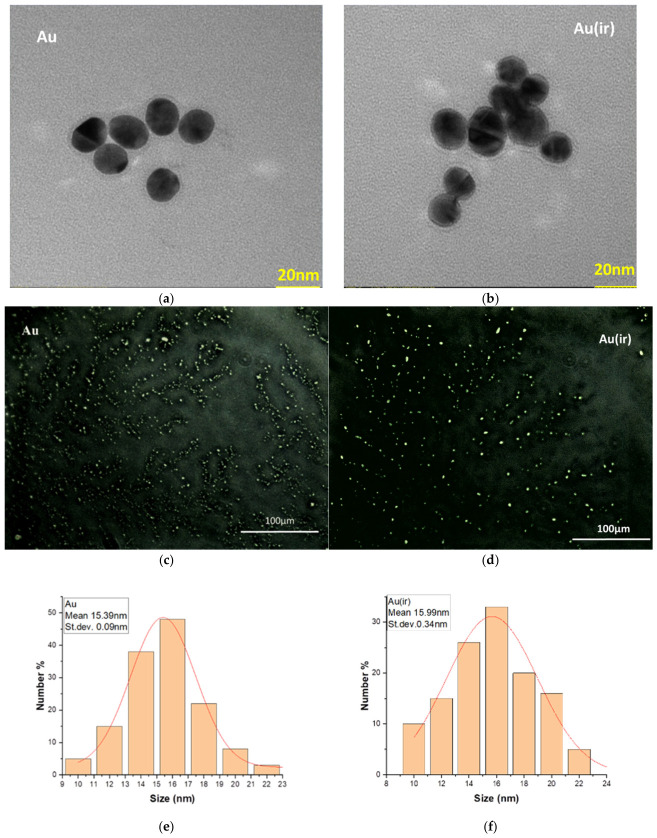
DF microscopy images for both the Au (**a**,**c**) and Au(ir) (**b**,**d**) samples and the size distributions of Au (**e**) and Au(ir) (**f**). The red line from graphs (**e**,**f**) represents the fitting of the distribution graphics.

It was observed that the average dimensions obtained for the Au and Au(ir) samples were close in value to the average dimensions of the same samples measured in TEM. The dimensional histograms obtained by ImageJ showed average values of 15.20 ± 0.008 nm for the measurement of 138 particles in the image of the Au sample (Figure 5e) and 15.99 ± 0.022 nm for 130 particles measured in the image of the Au(ir) sample (Figure 5f).

**Figure 6:**
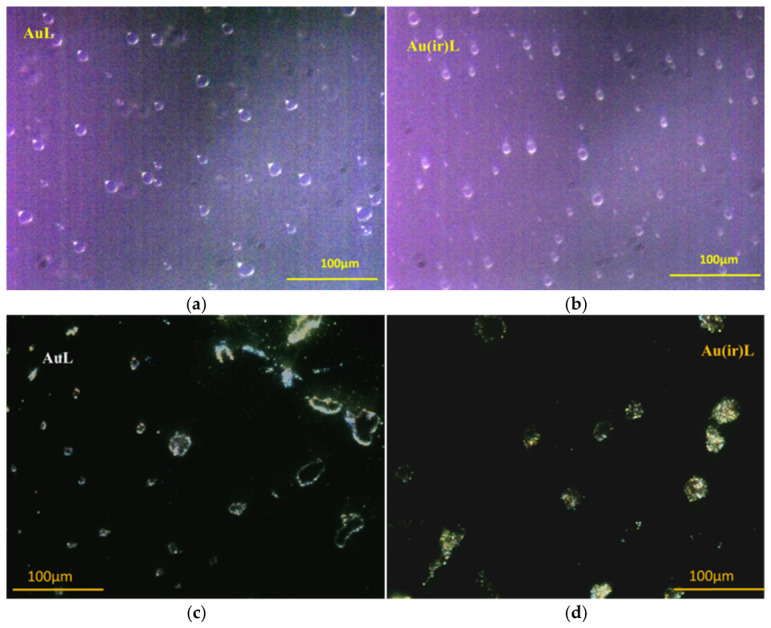
Images obtained by DF dark field microscopy performed on wet AuL sample (**a**), wet Au(ir)L sample (**b**), dry AuL (**c**) sample, and dry (**d**) Au(ir)L sample.

In addition, in the DF images, Figure 5b,d shows a tendency of the irradiated NPs to form dimeric groups compared to the non-irradiated ones, which were better dispersed. To observe how the nanoparticles were distributed upon interaction with the vesicles in both samples, DF images were taken on both the wet sample covered with the coverslip (Figure 6a,b) and on the sample not covered with the coverslip during drying using a 40× oil immersion objective. (Figure 6c,d). These DF images were taken on undiluted and unsonicated samples, which, near drying, began to disintegrate, highlighting the presence of nanoparticles trapped on the surface of the vesicle membrane without the slide being covered with the coverslip.

The images in Figure 6c show the AuL sample island formations obtained by joining the vesicles when drying; they appeared emptier and poorly grafted with nanoparticles. In the case of the Au(ir)L sample, according to Figure 6d, the island formations appeared to be abundantly grafted with nanoparticles. During the drying of the sample, the vesicles tended to merge, increasing in size and at the same time redistributing their nanoparticles on the surface of the lipid membrane [36].

#### 3.1.5. Hydrodynamic and Zeta Potential Dimension Analysis

To verify whether in the Au(ir)L sample the nanoparticles were better absorbed by the lipid bilayer of the vesicles than in the AuL sample as a result of the photooxidation of the citrate on the surface, hydrodynamic and zeta potential analysis were performed. The hydrodynamic size of the particles was determined in triplicate for all samples, and then the arithmetic mean was calculated.

**Figure 7:**
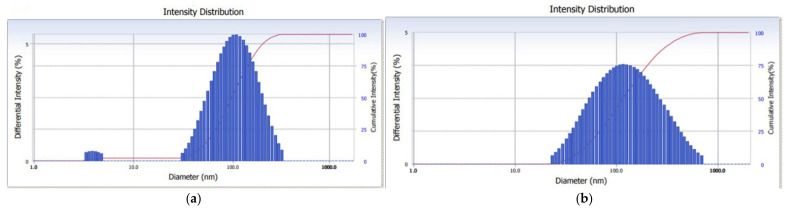
Dimensional histograms of the AuL (**a**) and Au(ir)L (**b**) samples. The red curve represents the Cumulative Intensity distribution (%).

The dimensional histogram of the AuL sample showed two species of particles, the first one is possibly due to AuNPs not captured on the vesicle surface, and the second one represents the vesicles to which nanoparticles are captured [37]. The dimensional histogram of the Au(ir)L sample showed a single species of particles, indicating the possibility that all irradiated nanoparticles were captured on the vesicle surface (Figure 7).

According to the hydrodynamic diameter measurements (Table 2) of the vesicles, the average values recorded for the AuL and Au(ir)L samples were 86.26 ± 2.4 nm and 103.23 ± 0.12 nm, respectively, corresponding to a polydispersity index of 0.259 and 0.261, respectively, indicating an increase in the degree of nanoparticle coverage of the vesicle surface in the case of the Au(ir)L sample compared to the AuL sample. The zeta potential measurements indicated negative values for AuL and Au(ir)L of −17.08 ± 2.5 mV and −23.8 ± 0.05 mV, respectively. An increase in the stability of the vesicles containing irradiated NPs was observed in the Au(ir)L sample, possibly due to the increase in the negative surface charge.

#### 3.1.6. FTIR Analysis

FTIR analysis was performed on the powder obtained from the suspensions deposited dropwise on glass Petri dishes. The suspension was dried at room temperature and then scraped, and the resulting powder was homogenized with sodium bromide and pressed to form a transparent pellet.

The three spectra carried out on the phosphatidylcholine lipid (L), Au L, and Au(ir)L samples reflect the influence of non-irradiated and irradiated gold nanoparticles on the functional groups of the lipid. The functional groups of the lipid are reflected in the characteristic vibrations as per the literature [38,39,40,41]. Thus, vibrations of free O–H at 3830 cm^−1^ and 3747 cm^−1^ and of bonded O–H at 3388 cm^−1^ come from the glycerol precursor or from water molecules adsorbed by the lipid molecule. The vibrations at 3388 cm^−1^ can also be attributed to the bonded N–H groups in choline. The C–H bonds in the lipid chain are found in the vibration at 3007 cm^−1^ for unsaturated groups (alkene type), while those in saturated groups (alkane type) are represented by vibrations at 2922 cm^−1^ and 2853 cm^−1^. The functional groups CH_2_ and CH_3_ are distinguished from each other by bending vibrations at 1455 cm^−1^ and 1380 cm^−1^, respectively. The unsaturation in the lipid chain is also reflected by the specific vibrations of the C=C double bond at 1645 cm^−1^ and 1547 cm^−1^ for stretching and at 821 cm^−1^ for bending. The ester carbonyl C=O groups are highlighted by the stretching vibration at 1736 cm^−1^ as well as the C–O groups with stretching vibrations at 1230 cm^−1^. Also at 1230 cm^−1^, the P=O groups vibrate, which, together with the vibrations at 1073 cm^−1^ assigned to the P–O groups, reflect the phosphate structure in the phosphatidylcholine molecule. The skeletal vibrations at 970 cm^−1^ are attributed to the methyl groups in the structure of choline N^+^(CH_3_)_3_.

**Figure 8:**
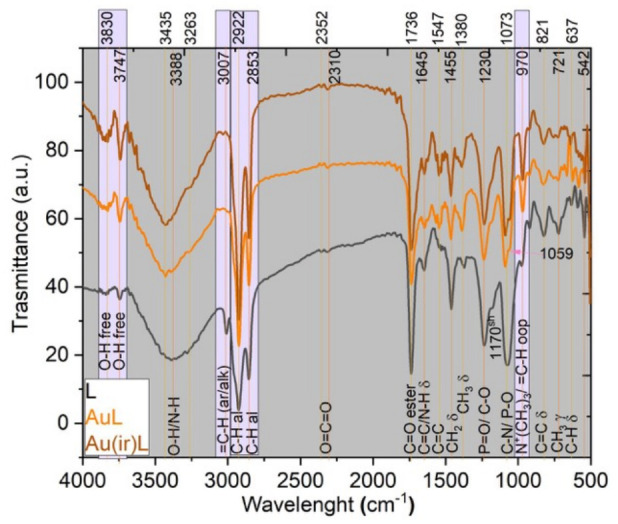
FTIR spectra of phosphatidylcholine—lipid (L), AuL, and Au(ir)L samples.

**Figure 9:**
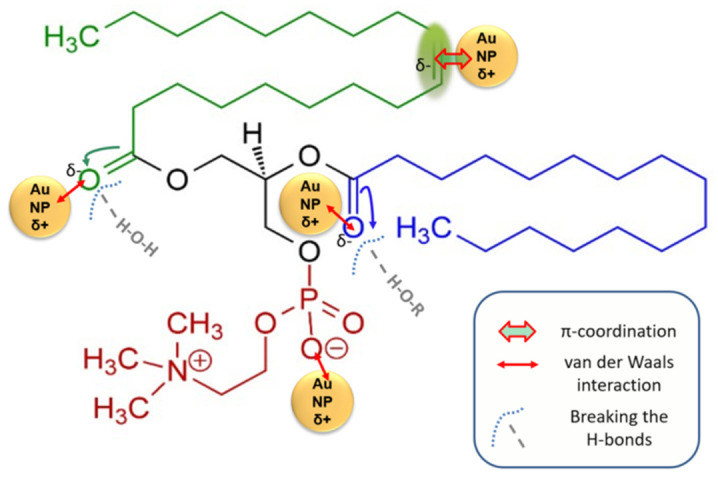
Schematic presentation of the mechanism assigned to the binding of gold nanoparticles to the phosphatidylcholine molecule.

Based on the changes resulting from the FTIR spectra (Figure 8), three mechanisms of binding of gold nanoparticles to the phosphatidylcholine molecule could be considered. The three mechanisms that we assume underlie the changes are presented schematically in Figure 9.

The first and most significant spectral change that distinguishes between simple lipid and lipid grafted with AuNPs is the absence of the peak at 3007 cm^−1^ in the AuL and Au(ir)L spectra. The absence of the peak indicates the interaction of AuNPs with the C=C bond in the lipid chain. AuNPs can act like Au^+^ ions. Thus, AuNPs would be attracted to the electron cloud of C=C. The interaction would be a π-coordination in which the carbon-carbon double bond donates electrons to the electrophilic gold ions. The result of such an interaction consists of stable Au–alkene complexes as agostic interactions C-H···Au that may contribute to building supramolecular structures in the form of polymers or gels. In this situation, the stretching vibrations of C-H bonds of the alkene type are replaced by those specific to alkanes, and by overlapping with those already present in the lipid not treated with AuNPs. This hypothesis can be supported by the increase in the peaks’ intensity at 2922 cm^−1^ and 2853 cm^−1^ [38,40,41] in the AuL and Au(ir)L spectra. This change in spectrum can confirm the binding of AuNPs to the lipid, also indicating the mechanism of attachment of metallic nanoparticles to the phosphatidylcholine molecule as well as the activation of the C-H bond. At the same time, the electronic cloud of C=C is still indicated by the specific vibrations in the AuL and Au(ir)L spectra.

The other spectral change compared to the lipid was at the level of free O-H groups (not involved in hydrogen bonds), in the sense that there was a significant increase in the intensity of the peaks at 3830 cm^−1^ and 3747 cm^−1^ for the AuL spectra and even more for Au(ir)L. This change can be explained by the fact that AuNPs, and especially irradiated AuNPs, promote their van der Waals-type electrostatic interactions with the C=O groups of the lipid by removing the O-H groups hydrogen-bonded to them [37,38,39,40]. This change can be explained by the fact that AuNPs, and especially irradiated AuNPs, promote their van der Waals electrostatic interactions with the C=O groups of the lipid by removing the O-H groups of water or traces of glycerol bound by hydrogen bonds to the ester carbonyls of phosphatidylcholine. This modification in the AuL and Au(ir)L spectra compared to that of the lipid may indicate the attachment of AuNPs to phosphatidylcholine and at the C=O groups, with an increase in interaction in the case of irradiated nanoparticles.

The 970 cm^−1^ skeletal vibration assigned to methyl group in N^+^(CH_3_)_3_ [36] shows an intensification in the AuL and Au(ir)L spectra compared to the lipid, indicating the involvement of the negative pole of the phosphatidylcholine molecule (phosphate group) in an interaction with AuNPs, while the positive pole of the molecule can participate in intra- and intermolecular interactions.

#### 3.1.7. SEM-EDX Analysis

**Figure 10:**
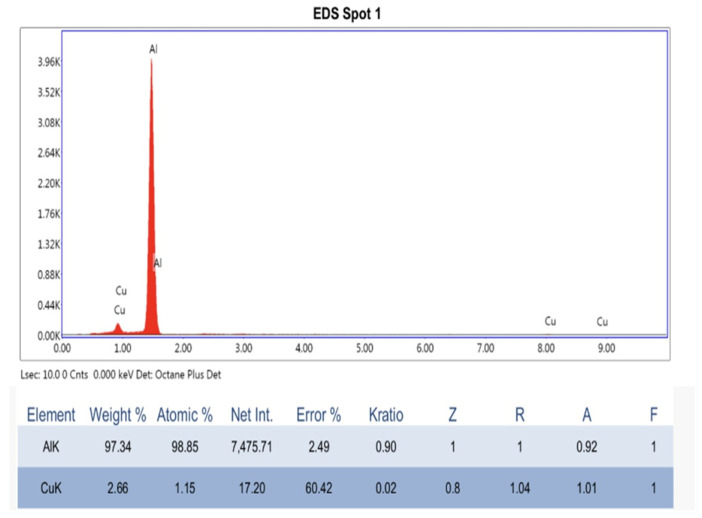
EDX spectrum of the aluminum substrate.

In order to explain the stronger interaction of the irradiated gold nanoparticles with the lipid bilayer, SEM-EDX analysis was performed on 4 different points of each sample studied. The table below contains the average values of the mass and atomic concentrations for each sample analyzed. The Al signal in the EDX data originates from the aluminum substrate used for SEM sample deposition (Figure 10).

As per Table 3, an increase in the atomic concentration of Au from 0.027% in the AuL sample to 0.04% in the Au(ir)L sample was observed. On the other hand, the Au(ir)L sample recorded a decrease in the atomic concentration of PK from 2.25% in AuL to 0.25% in Au(ir)L and of Nk from 1.48% in AuL to 0.52% in Au(ir)L. The decrease in the concentration of phosphorus and nitrogen in the case of Au(ir)L suggests a strong availability of nanoparticles to attach to the hydrophilic head of the vesicle membrane due to the increase in the level of interaction of phosphatidylcholine with the gold nanoparticles. The disappearance of Na and Cl ions was also observed in the case of the Au(ir)L sample. The high Al content of the Au(ir)L sample (76.37 wt%) reflects a thinner layer of sample over the layer, rather than aluminum in the formulation itself.

Dimeric structures are formed at the membrane surface by van der Waals interactions between adjacent nanoparticles adsorbed on the same vesicle and do not represent aggregation of free particles in suspension. The overall colloidal stability of the Au(ir)L system is conferred by the increased negative surface charge (ζ = −23.8 mV), which prevents inter-vesicle aggregation. According to a kinetic model of the aggregation of gold nanoparticles on the surface of vesicles, depending on the electrolyte concentration, it is considered that upon trapping AuNPs, most of the adsorbed anions are released, reducing their negative potential and becoming practically neutral.

**Figure 11:**
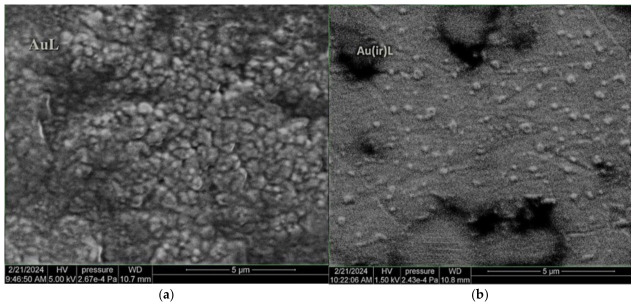
SEM images of the AuL (**a**) and Au(ir)L (**b**) samples.

The van der Waals component of these NPs becomes more significant, leading to dimeric formations of AuNPs on the vesicle surface [34]. The results of the elemental analysis and their interpretation are consistent with the FTIR analysis results previously presented (Section 3.1.6). In Figure 11, the image acquired with the electronic microscopy technique showed vesicle clusters for the AuL sample (a) compared to dispersed vesicles for the Au(ir)L sample (b). The scattered vesicles in the Au(ir)L sample could be associated with the breaking of hydrogen bonds reported in the FTIR spectra analysis. Since the dispersion medium in Figure 11b must also be taken into account, where it is clearly observed as a continuous matrix, based on the same FTIR spectra, a polymerization of the lipid or a gelling of it can also be considered, in which unpolymerized vesicles or nanoparticle accumulation centers are formed. This structure provides greater stability to lipid vesicles grafted with gold nanoparticle systems and confirms an improvement in the grafting mechanism through AuNP irradiation.

### 3.2. In Vivo Study

#### 3.2.1. Effects on Zebrafish Anxiety Response in the NTT

**Figure 12:**
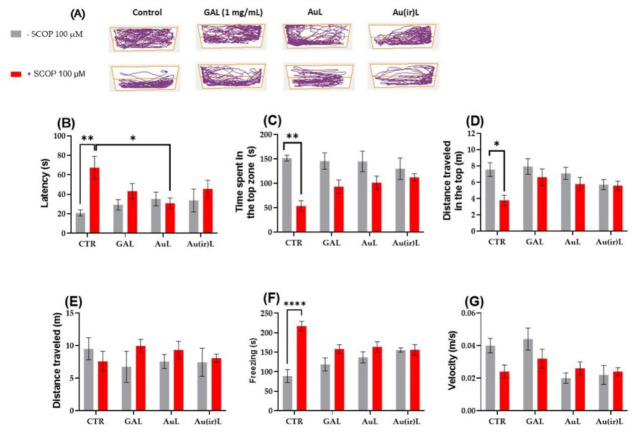
Behavioral effects of lipid vesicles grafted with gold nanoparticles in zebrafish subjected to the NTT. Naive zebrafish and those treated with scopolamine (SCOP, 100 μM) were exposed to AuL and Au(ir)L. Galantamine (GAL, 1 mg/L) served as the positive control. (**A**) Representative swimming trajectories during the 6-min test; (**B**) Latency to first entry into the top zone; (**C**) Time spent in the top zone (s); (**D**) Distance traveled in the top zone (m); (**E**) Total distance traveled (m); (**F**) Freezing duration (s); (**G**) Average swimming speed (m/s). Data are presented as the mean ± SEM (*n* = 6 per group). Statistical analysis was performed using one-way ANOVA followed by Tukey’s post hoc test. * *p* < 0.01, ** *p* < 0.001, and **** *p* < 0.00001.

To evaluate the anxiolytic or anxiogenic effects of lipid vesicles grafted with irradiated gold nanoparticles, zebrafish were exposed to AuL and Au(ir)L. The behavioral response was assessed in both untreated (naïve) fish and those pretreated with SCOP using the NTT. During the 6 min test, primary anxiety-related parameters were recorded: latency to enter the top zone (s), time spent in the top zone (s), and distance traveled in the top zone (m) (Figure 12B–D). To further characterize the neurobehavioral phenotype associated with anxiety-like behavior, the following secondary parameters were also analyzed: total distance traveled (m), freezing duration (s), and average swimming velocity (m/s) (Figure 12E–G). In the NTT, an extended 6 m observation using representative locomotion tracking maps revealed distinct swimming behaviors across experimental groups (Figure 12A). Zebrafish in the CTR and GAL groups exhibited a clear preference for the top zone of the tank, reflecting a typical exploratory pattern and reduced anxiety. In contrast, zebrafish exposed to SCOP (100 μM) demonstrated a significant increase in bottom-dwelling behavior, indicative of an anxiety-like state commonly associated with SCOP administration.

Regarding acute treatments with AuL and Au(ir)L, no significant deviations in baseline behavior were observed compared to the CTR group. However, in SCOP co-treated groups, both AuL and Au(ir)L showed a slight increase in time spent and distance traveled in the top zone compared to SCOP alone, suggesting a partial anxiolytic effect similar to that observed in the SCOP + GAL group.

Tukey’s post hoc analysis revealed significant intergroup differences across several behavioral parameters. For latency to enter the top zone, SCOP treatment resulted in increased values relative to the CTR group (*p* < 0.01, Figure 11b, reflecting delayed exploratory behavior. Notably, co-treatment with SCOP + AuL and SCOP + Au(ir)L significantly reduced latency compared to SCOP alone (*p* < 0.01, Figure 12B), indicating a potential anxiolytic-like effect of these formulations.

Time spent in the top zone, a key indicator of reduced anxiety, was markedly decreased in the SCOP group (29.5–86.9 s) relative to the CTR group (135.1–166.4 s; *p* < 0.001, Figure 12C). Although the SCOP + AuL and SCOP + Au(ir)L groups exhibited slightly elevated values, these improvements were not statistically significant compared to SCOP alone.

Distance traveled in the top zone followed a similar trend. While the SCOP group showed a significant reduction (*p* < 0.01, Figure 12D) compared to CTR, the SCOP + AuL and SCOP + Au(ir)L groups did not significantly differ from the SCOP group (Figure 12D). However, a trend toward recovery was observed.

A comprehensive analysis of additional locomotor parameters, including total distance, freezing, and velocity (Figure 12E–G), further confirmed the anxiogenic effects of SCOP. Compared to CTR, SCOP-treated zebrafish exhibited significantly reduced total distance and velocity, alongside increased freezing duration. While AuL and Au(ir)L moderately improved these parameters (Figure 12E–G), only SCOP + Au(ir)L showed statistically significant reductions in freezing duration (*p* < 0.01, Figure 12F), suggesting enhanced anxiolytic and locomotor-restorative effects of AuL nanoparticles.

#### 3.2.2. Effects on Zebrafish Spatial Memory in the Y-Maze

**Figure 13:**
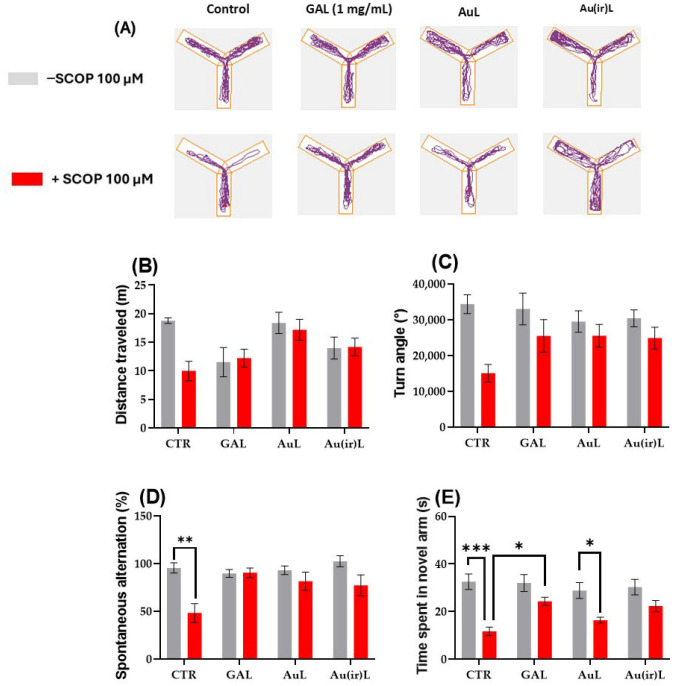
Behavioral effects of lipid vesicles grafted with gold nanoparticles in zebrafish subjected to the Y-maze. Naive zebrafish and those treated with scopolamine (SCOP, 100 μM) were exposed to AuL and Au(ir)L. Galantamine (GAL, 1 mg/L) served as the positive control. (**A**) Representative swimming trajectories during the Y-maze test; (**B**) Total distance traveled (m) in different groups; (**C**) Turn angle (°) in different groups; (**D**) Spontaneous alternations (%) in different groups; (**E**) Time spent in the novel arm (s) in different groups. Data are presented as the mean ± SEM (*n* = 6 per group). Statistical analysis was performed using one-way ANOVA followed by Tukey’s post hoc test. * *p* < 0.01, ** *p* < 0.001, and *** *p* < 0.0001.

Figure 13A illustrates the locomotor behavior of zebrafish in the Y-maze test, revealing distinct differences in spatial memory performance among the experimental groups. Zebrafish exposed to SCOP (100 μM) spent significantly more time in the other arm and less time in the novel arm compared to the controls. This behavioral pattern reflects a marked impairment in spatial recognition memory and suggests that SCOP induces deficits in both recall and discrimination of previously explored environments. Tukey’s post hoc analyses revealed no significant differences in the total distance traveled across the experimental groups, indicating that general locomotor activity remained consistent (Figure 13B). However, zebrafish treated with SCOP exhibited a significant reduction in turn angle compared to the CTR group (*p* < 0.01, Figure 13B), reflecting impaired navigational and exploratory behaviors. Notably, co-treatment with SCOP + AuL and SCOP + Au(ir)L significantly improved turn angle relative to SCOP alone (*p* < 0.01, Figure 13B).

Spatial memory and exploratory performance were assessed using spontaneous alternation percentage and time spent in the novel arm of the Y-maze. As shown in Figure 13D, spontaneous alternation percentage was robust in the CTR group (77–110 entries), while SCOP treatment led to a marked reduction (27–79 entries; *p* < 0.001, Figure 13D), indicating impaired spatial memory. A similar pattern was observed in the time spent in the novel arm, which significantly declined under SCOP treatment (6.17–15 s) compared to CTR (21.77–39.53 s; *p* < 0.0001, Figure 13E).

GAL-treated fish maintained high levels of spontaneous alternation percentage (+SCOP: 75–100) and increased time spent in the novel arm (22.67–29 s; *p* < 0.01), confirming its cognitive-enhancing effects. In the AuL-treated group, spontaneous alternation percentage remained moderate (+SCOP: 58–100), with a modest improvement in time spent in the novel arm (14–19.97 s) (*p* < 0.01), although this effect was not statistically significant (Figure 13E). Au(ir)L produced slightly better outcomes, with more consistent alternation scores (+SCOP: 57–108) and improved novel arm exploration (+SCOP: 15.93–29.77 s), again without reaching significance.

#### 3.2.3. Effects on Acetylcholinesterase (AChE) Activity

AChE is a key enzyme in the regulation of cholinergic neurotransmission, responsible for hydrolyzing acetylcholine (ACh) in the synaptic cleft. Elevated AChE activity is associated with reduced synaptic ACh levels and is often linked to cognitive impairments [42]. In this study, the specific activity of AChE was measured in the brains of zebrafish exposed to SCOP, a muscarinic antagonist commonly used to induce cognitive deficits.

**Figure 14:**
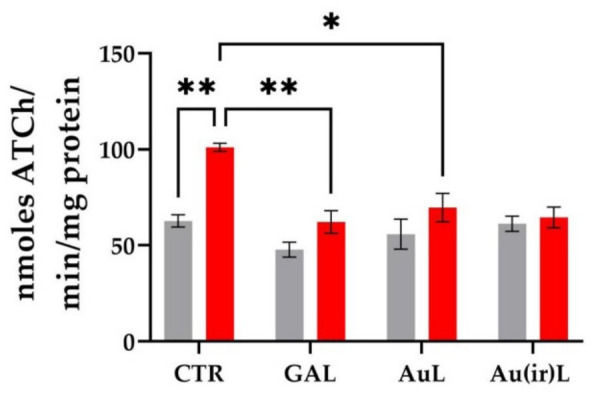
Effects of lipid vesicles grafted with gold nanoparticles on acetylcholinesterase (AChE) activity in zebrafish exposed to scopolamine (SCOP, 100 μM). AChE activity following treatment with AuL and Au(ir)L. Data are presented as the mean ± SEM (*n* = 3). Statistical significance was determined using Tukey’s post hoc test: * *p* < 0.05, and ** *p* < 0.01. The gray bars correspond to −SCOP and the red bars correspond to +SCOP.

In the CTR group, AChE activity remained within physiological limits (59–69 units), reflecting normal enzymatic function. In contrast, zebrafish treated with SCOP exhibited significantly elevated AChE activity (97–105 units, *p* < 0.001, Figure 14), indicating enzyme hyperactivation following cholinergic disruption. Tukey’s post hoc analysis confirmed a statistically significant increase in AChE activity in the SCOP group compared to the controls (*p* < 0.001, Figure 14).

Treatment with GAL, a well-established AChE inhibitor [43], resulted in a marked reduction in AChE activity under both baseline and SCOP-exposed conditions. AChE activity in the GAL-treated group ranged from 41–54 units (−SCOP) and 51–71 units (+SCOP), indicating effective inhibition of the enzyme. Significant differences were also observed between the SCOP-only and SCOP + GAL groups (*p* < 0.001, Figure 14), supporting the therapeutic role of GAL in modulating cholinergic function under cognitive impairment conditions. The effects of AuL and Au(ir)L on AChE activity in zebrafish exposed to SCOP are shown in Figure 14. Treatment with AuL resulted in a significant reduction in AChE activity (40.42–83.99 units) (*p* < 0.01) compared to the SCOP-only group, suggesting an inhibitory effect. Moreover, Au(ir)L significantly improved AChE modulation, leading to a notable reduction in enzyme activity compared to SCOP (*p* < 0.01). These results indicate that irradiation enhances the neuroprotective efficacy of liposomal gold nanoparticles, potentially by improving their stability or bioavailability.

#### 3.2.4. Effects on Oxidative Stress Markers

**Figure 15:**
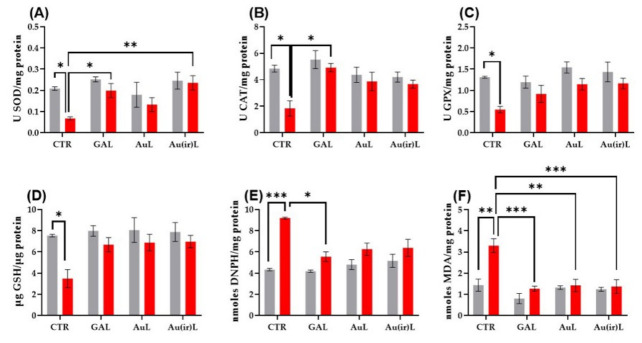
Effects of lipid vesicles grafted with gold nanoparticles on zebrafish brain oxidative stress markers: (**A**) SOD, (**B**) CAT, and (**C**) GSH specific activities, (**D**) GPX, (**E**) carbonylated proteins, and (**F**) MDA levels. Values are expressed as the mean ± SEM (*n* = 3). Tukey post hoc analyses revealed significant statistical differences as follows: * *p* < 0.05, ** *p* < 0.01, and *** *p* < 0.001. The gray bars correspond to −SCOP and the red bars correspond to +SCOP.

SOD plays a key role in the antioxidant defense system by catalyzing the dismutation of superoxide anions into hydrogen peroxide and molecular oxygen, thereby protecting cells from oxidative damage [44]. In this study, gold-based nanoparticles enhanced SOD activity in zebrafish subjected to SCOP-induced oxidative stress. Notably, Tukey’s post hoc analysis revealed a significant increase in SOD activity in the SCOP + Au(ir)L group compared to the SCOP-only group (*p* < 0.001, Figure 15A), while non-irradiated AuL showed no significant effect.

CAT activity, which contributes to oxidative stress mitigation by decomposing hydrogen peroxide into water and oxygen [45], was also evaluated. Although CAT activity increased slightly following treatment with AuL and Au(ir)L, statistical significance was not reached in either case (Figure 15B), suggesting limited efficacy of these formulations in restoring CAT function under SCOP exposure.

GPX, which reduces hydrogen peroxide and lipid peroxides using GSH as a cofactor [46], was not significantly modulated by either AuL or Au(ir)L (Figure 15E), indicating that these formulations may not effectively influence GPX-mediated antioxidant defense mechanisms.

Similarly, GSH content, a critical component of cellular redox balance, remained unaffected by both AuL and Au(ir)L treatment (Figure 15D), suggesting a limited impact on the endogenous antioxidant reservoir.

In contrast, protein carbonylation, a marker of oxidative protein damage [47], was significantly reduced following treatment with both AuL and Au(ir)L (*p* < 0.01, Figure 15E).

MDA levels, a key indicator of lipid peroxidation, were significantly reduced in the SCOP + AuL (*p* < 0.001, Figure 15F) and SCOP + Au(ir)L (*p* < 0.0001, Figure 15F) groups, confirming a protective effect against membrane lipid damage. These findings indicate that AuL nanoparticles, particularly in their irradiated form, possess moderate antioxidant potential, primarily by reducing superoxide burden and inhibiting lipid peroxidation.

The modulation of antioxidant enzyme activities (SOD, CAT, and GPX), together with the reduction in lipid peroxidation, supports the antioxidant effect of the nanoparticle formulations, indicating their ability to enhance endogenous antioxidant defense mechanisms and mitigate oxidative stress at the tissue level.

## 4. Discussion

In this study, lipid vesicles grafted with both non-irradiated gold nanoparticles and gold nanoparticles irradiated under green light were made. The images obtained by DF microscopy detected differences between these vesicles and showed an increase in the interaction of irradiated nanoparticles with lipid vesicles in the Au(ir)L sample compared to the AuL sample. The UV–Vis spectra confirmed an increase in the LSPR band in the Au(ir)L sample, but also a red shift compared to the AuL sample. According to the TEM micrograph, irradiation of the nanoparticle suspension did not lead to significant changes in the size of the gold nanoparticles, Dark field microscopy made it possible to observe some differences between the AuL and Au(ir)L samples by gradual drying. In the image of the AuL sample, the vesicles appeared to have rare gold nanoparticles trapped on the surface of the lipid bilayer. In the Au(ir)L sample, the vesicles appeared to be more abundantly covered with gold nanoparticles. A series of indirect analyses seemed to confirm the observations obtained by dark-field optical microscopy. Increases in hydrodynamic size and zeta potential were recorded in the Au(ir)L sample compared to the AuL sample. The local increase in negative charge at the vesicle surface in the Au(ir)L sample allowed for an increase in stability and colloidal dispersion through electrostatic repulsion [44]. According to other studies, the increase in zeta potential may depend on the absorption of a larger number of nanoparticles decorating the liposome/vesicle [44].

SEM-EDX analysis indicates an increase in the interaction of irradiated nanoparticles with the hydrophilic head of lipid molecules in the vesicle membranes, this aspect being recorded in reports from the specialized literature that showed that the main mode of interaction between gold nanoparticles and the lipid membrane in liposomal models as being through the polar head of lipids [37,45]. However, based on the FTIR analysis, three mechanisms by which gold nanoparticles can attach to the lipid molecule were assigned. Spectral changes indicate three binding modes, namely π-coordination, as well as van der Waals interactions at the phosphate group and carbonyl groups. In the latter case, the binding of nanoparticles by van der Waals forces to the double oxygen bonded in C=O led to the removal of water molecules and/or glycol molecules that previously interacted with the lipid through hydrogen bonds. We assume that green light irradiation induced partial photooxidation of the citrate layer on the AuNP surface, reducing steric repulsion and facilitating electrostatic interaction with phospholipid groups. This hypothesis is consistent with the presented spectral, morphological, and physicochemical data, but requires direct validation by surface-sensitive techniques such as XPS or quantitative binding assays, which will be addressed in future work.

An in vivo study was performed on a zebrafish model.

The current study demonstrates that both AuL and irradiated Au(ir)L nanoparticles exhibit promising neuroprotective properties in a zebrafish model of SCOP-induced cognitive impairment. It is important to note that SCOP was administered as a pretreatment step, and fish were subsequently exposed to nanoparticle formulations in fresh media. This experimental design minimizes the likelihood of direct interaction between scopolamine and the nanoparticles. Additionally, given the physicochemical properties of SCOP and the stabilized nature of the lipid vesicles grafted with gold nanoparticles, significant direct binding interactions are unlikely. Importantly, the lack of significant differences between the control and nanoparticle-treated groups under non-SCOP conditions suggests that the formulations do not exert off-target behavioral or biochemical effects, supporting their biocompatibility at the tested concentrations. Both AuL and Au(ir)L showed differences in physicochemical properties, as evidenced by spectrophotometric, zeta potential, and SEM-EDX analyses, the functional significance of which on biological outcomes remains to be fully established. In behavioral assessments, both formulations showed partial anxiolytic effects in the NTT, with reductions in the latency to enter the upper zone under SCOP treatment for both AuL and Au(ir)L. A trend toward shorter freezing duration was observed for Au(ir)L without demonstrating consistent statistical superiority of Au(ir)L over AuL in all behavioral assessment criteria. Although several behavioral improvements were observed following treatment with both nanoparticle formulations, not all parameters reached statistical significance. However, consistent trends across multiple endpoints, together with statistically significant effects observed on key behavioral and biochemical markers, suggest a biologically relevant neuroprotective effect for both formulations. In the Y-maze test, SCOP-treated fish exhibited pronounced spatial memory deficits. Both AuL and Au(ir)L showed trends toward improved rotation angle compared to SCOP alone, although the differences between the two formulations did not reach statistical significance. At the biochemical level, both AuL and Au(ir)L produced statistically significant reductions in AChE activity compared to the SCOP group, with comparable effects between the two formulations, suggesting that both may modulate cholinergic function under conditions of cognitive impairment. Oxidative stress markers further supported the neuroprotective potential of both formulations. Au(ir)L significantly increased SOD activity, consistent with the reduction in superoxide load, whereas AuL did not reach significance for this marker. Although neither formulation significantly modulated CAT, GPX, or GSH content, both AuL and Au(ir)L significantly reduced the MDA levels to a comparable extent, indicating equivalent protection against lipid peroxidation. The observed behavioral improvements may be mechanistically related to the biochemical changes induced by both nanoparticle formulations. The reduction in AChE activity suggests an increase in cholinergic neurotransmission, while the modulation of oxidative stress markers indicates attenuation of oxidative damage in the brain. Overall, the data indicate that AuL and Au(ir)L exhibit comparable efficacy in most behavioral and biochemical endpoints, despite differences in their physicochemical characteristics. The potential advantages of irradiation on biological efficacy require confirmation in future studies with larger samples and dose–response models.

Notably, Au(ir)L demonstrated superior efficacy, likely due to enhanced physicochemical stability and improved interaction with the lipid bilayer, as supported by spectrophotometric, zeta potential, and SEM-EDX analyses. In behavioral assessments, both AuL and Au(ir)L showed partial anxiolytic effects in the NTT, with a reduction in latency to enter the top zone under SCOP treatment. Au(ir)L showed a reduction in freezing duration; however, this effect was not consistently statistically significant.

Both AuL and Au(ir)L showed comparable effects across behavioral and biochemical parameters, with no clear evidence of superiority for either formulation.

In the Y-maze test, SCOP-treated fish exhibited pronounced spatial memory deficits, including reduced spontaneous alternation percentage and time spent in the novel arm. Au(ir)L showed trends toward improvement in turn angle and spontaneous alternation; however, these effects were not statistically significant.

No clear differences between AuL and Au(ir)L were observed for these parameters.

Both AuL and Au(ir)L reduced AChE activity compared to SCOP-treated groups, with no clear difference in magnitude between the two formulations. This suggests a superior ability of Au(ir)L to preserve cholinergic function, potentially contributing to its enhanced behavioral efficacy. Oxidative stress markers further supported the neuroprotective potential of these formulations. Au(ir)L significantly increased the SOD activity, consistent with reduced superoxide burden. Although neither AuL nor Au(ir)L significantly modulated CAT, GPX, or GSH content, Both AuL and Au(ir)L significantly reduced the MDA levels to a similar extent based on Figure 15. The behavioral improvements observed in the present study can be mechanistically linked to the biochemical changes induced by the nanoparticle formulations. The reduction in AChE activity suggests an enhancement of cholinergic neurotransmission, which is essential for learning and memory processes and is known to be impaired in scopolamine-induced cognitive dysfunction. In parallel, the modulation of oxidative stress markers, particularly the increase in SOD activity and the reduction in MDA and protein carbonyl levels, indicates an attenuation of oxidative damage in the brain. Since oxidative stress is closely associated with both cognitive deficits and anxiety-like behavior, these biochemical effects likely contribute to the observed improvements in behavioral performance. Au(ir)L exhibited effects comparable to those observed for AuL. These findings suggest that the antioxidative capacity of Au(ir)L is primarily exerted through the SOD pathway and membrane protection. Au(ir)L and AuL showed broadly similar neuroprotective effects, despite differences in their physicochemical properties and interaction with lipid vesicles. This aligns with the spectroscopic and morphological findings that demonstrated higher lipid coverage and negative surface charge for Au(ir)L compared to AuL.

The present study has several limitations that should be acknowledged. First, the absence of additional control groups, such as free AuNPs and lipid vesicles without nanoparticles, limits the ability to disentangle the individual contributions of each component and to fully assess potential synergistic effects. Inclusion of these groups in future studies would provide a more comprehensive understanding of the mechanisms underlying the observed neuroprotective activity. In addition, the relatively small sample size may reduce statistical power and limit the detection of subtle effects. Although the number of animals used is consistent with previously reported zebrafish studies employing similar behavioral paradigms, increasing the sample size in future investigations would enhance the robustness and reliability of the findings.

Furthermore, the absence of dose–response studies and the relatively short exposure duration limit the interpretation of the full therapeutic potential of the formulations. Future studies should include multiple dose levels and extended treatment protocols to better characterize efficacy and long-term effects.

## 5. Conclusions

Lipid vesicles grafted with non-irradiated and green light-irradiated gold nanoparticles were obtained. The characterization of these vesicles showed that the use of irradiated nanoparticles led to their much more abundant absorption on the vesicle surface than that of non-irradiated nanoparticles. This study provides initial evidence that both AuL and Au(ir)L nanoparticles exhibit promising neuroprotective properties in a zebrafish model of SCOP-induced cognitive dysfunction. Both formulations showed trends toward anxiolytic and cognition-supporting effects, with several statistically significant improvements in behavioral and biochemical endpoints. Although Au(ir)L exhibited distinct physicochemical characteristics—including higher lipid coverage, more negative zeta potential, and preferential SOD activation—it did not show statistically significant superiority over AuL in all endpoints. In particular, both formulations significantly reduced the AChE activity and MDA levels to comparable extents. Given their general biocompatibility and multi-targeting potential, lipid-grafted gold nanoparticles represent a promising platform for further investigation in neurodegenerative disorders characterized by cholinergic deficits and oxidative stress. Future studies with larger sample sizes, dose–response models, and expanded treatment protocols are needed to fully characterize the therapeutic potential and determine whether irradiation confers a consistent biological advantage.

This study provides compelling evidence that AuL and Au(ir)L) nanoparticles possess neuroprotective properties in a zebrafish model of SCOP-induced cognitive dysfunction. While both formulations exhibited anxiolytic and cognition-enhancing effects, Au(ir)L consistently outperformed AuL, particularly in reversing SCOP-induced deficits in memory and exploratory behavior. The superior efficacy of Au(ir)L appears to stem from its enhanced physicochemical properties, including increased lipid bilayer integration, greater electrostatic stability, and improved antioxidant effects, especially in reducing superoxide radicals and lipid peroxidation. Moreover, the ability of Au(ir)L to normalize AChE activity underlines its potential as a modulator of cholinergic transmission. Given their biocompatibility and multi-target actions, irradiated lipid vesicles grafted with gold nanoparticles emerge as promising nanotherapeutic agents for neurodegenerative conditions characterized by cholinergic deficits and oxidative stress. While these findings are promising, further studies are required to confirm these effects under extended exposure conditions and across different dosing regimens.

## Figures and Tables

**Table 1:** Absorbance and concentration of aged nanoparticles.

Sample	λmax	Absorbance (A)	Concentration
AuNP	522	Y = 0.67	3.333 nM
Au(ir)	523	Y = 0.92	4.577 nM
AuL fresh	526	Y = 1.1949	5.945 nM
Au(ir)L fresh	529	Y = 1.2557	6.247 nM
AuL aged	527	Y = 0.68	3.40 nM
Au(ir)L aged	527.5	Y = 0.7043	3.50 nM

**Table 2:** Evaluation of hydrodynamic size and zeta potential for the analyzed samples.

Sample	Hydrodynamic Dimension (nm)			Polydispersity Index			Zeta Potential (mV)		
AuL	89.4	83.3	86.1	0.248	0.271	0.259	−20.2	−14.06	−16.98
	86.26			0.259			−17.08		
Au(ir)L	103.1	103.4	103.2	0.260	0.259	0.266	−24.13	−23.84	−23.45
	103.23			0.261			−23.8		

**Table 3:** Atomic and molecular concentration of chemical elements on the surface of vesicles.

Sample	Element	Weight %	Atomic %	Error %
AuL	C_K_	31.81	42.52	0.06
	N_K_	1.27	1.48	0
	O_K_	33.85	35.07	0.05
	Na_K_	4.37	3.15	0.01
	P_K_	4.18	2.25	0.02
	Au_M_	0.3	0.027	0
	Cl_M_	0.75	0.38	0.01
	Al_K_	23.47	15.12	0.55
Au(ir)L	C_K_	17.58	27.33	0.01
	N_K_	0.3	0.52	0
	O_K_	5.43	7.24	0.01
	Na_K_			
	P_K_	0.49	0.25	0
	Au_M_	0.3	0.04	0
	Cl_K_			
	Al_K_	76.37	65.03	0.48

## Data Availability

The original contributions presented in this study are included in the article. Further inquiries can be directed to the corresponding authors.
